# Respiratory Infections Following Earthquake-Induced Tsunamis: Transmission Risk Factors and Lessons Learned for Disaster Risk Management

**DOI:** 10.3390/ijerph18094952

**Published:** 2021-05-06

**Authors:** Maria Mavrouli, Spyridon Mavroulis, Efthymios Lekkas, Athanassios Tsakris

**Affiliations:** 1 Department of Microbiology, Medical School, National and Kapodistrian University of Athens, 11527 Athens, Greece; atsakris@gmail.com; 2 Department of Dynamic Tectonic Applied Geology, Faculty of Geology and Geoenvironment, School of Sciences, National and Kapodistrian University of Athens, 15784 Athens, Greece; smavroulis@geol.uoa.gr (S.M.); elekkas@geol.uoa.gr (E.L.)

**Keywords:** tsunami, polymicrobial infection, tsunami lung, influenza, pneumonia, transmission risk factors, emergency shelters, evacuation centers, disaster risk management

## Abstract

Earthquake-induced tsunamis have the potential to cause extensive damage to natural and built environments and are often associated with fatalities, injuries, and infectious disease outbreaks. This review aims to examine the occurrence of respiratory infections (RIs) and to elucidate the risk factors of RI transmission following tsunamis which were induced by earthquakes in the last 20 years. Forty-seven articles were included in this review and referred to the RIs emergence following the 2004 Sumatra-Andaman, the 2009 Samoa, and the 2011 Japan earthquakes. Polymicrobial RIs were commonly detected among near-drowned tsunami survivors. Influenza outbreaks were commonly detected during the influenza transmission period. Overcrowded conditions in evacuation centers contributed to increased acute RI incidence rate, measles transmission, and tuberculosis detection. Destruction of health care infrastructures, overcrowded evacuation shelters, exposure to high pathogen densities, aggravating weather conditions, regional disease endemicity, and low vaccination coverage were the major triggering factors of RI occurrence in post-tsunami disaster settings. Knowledge of risk factors underlying RIs emergence following earthquake-induced tsunami can contribute to the implementation of appropriate disaster prevention and preparedness plans characterized by sufficient environmental planning, resistant infrastructures, resilient health care facilities, and well-established evacuation centers. Global and local disease surveillance is a key prerequisite for early warning and protection against RIs’ emergence and transmission in tsunami-prone areas.

## 1. Introduction

A tsunami is a gravity wave, or series of gravity waves, generated when a large volume of water is vertically or horizontally displaced by a sudden disturbance [1,2,3]. More than 85% of tsunamis have tectonic causes, such as earthquake-induced fault movements, and more than 80% of them have occurred in the Pacific Ocean [4,5].

Tsunamis have a disastrous impact, not only on the natural (i.e., coastal geomorphological changes, soil erosion) and built environment of the affected coastal areas, but also on public health [6,7,8,9,10]. Thus, tsunamis are often associated with fatalities, injuries, infectious disease outbreaks, and mental health issues. Mass fatalities during acute incidences mainly occur due to drowning, burying by tsunami deposits left onshore or offshore, entrapment inside collapsing buildings, and severe traumatic injuries during phases of uprush during inundation of coastal areas and backwash when tsunami water recedes [11,12,13,14].

Tsunamis may also cause severe mental health problems, such as severe, mild, or moderate mental disorders (e.g., psychosis, depression, anxiety disorders) and psychological distress [15,16].

Infectious diseases commonly emerge following disasters induced by natural hazards [17]. Tsunami survivors are at increased risk of respiratory infections (RIs), which may occur either from the direct exposure to the microbial flora of the aspirated tsunami water or as a result of the post-tsunami settings in affected areas.

Regarding infectious diseases related to tsunamis, although there are several reports or studies in the literature, respiratory infectious diseases (RIDs) associated with tsunamis have not been investigated in a systematic review process. The current study involved an extensive and systematic literature review aiming to assess the impact of earthquake-induced tsunamis to public health, and more specifically, to examine the occurrence of potential RIs following earthquake-induced tsunamis and to elucidate the risk factors that predispose tsunami survivors to RIs in the tsunami’s aftermath.

## 2. Methods

### Search Strategy

All major databases and sources for scientific, technical, and medical research contained in the National Center for Biotechnology Information (NCBI, https://www.ncbi.nlm.nih.gov/, accessed on 19 October 2020), part of the United States National Library of Medicine (NLM), were thoroughly searched in October 2020 to identify documented RIs in humans occurring worldwide from January 2000 to January 2020, where an earthquake-induced tsunami was believed to have been involved. More specifically, key word searches were conducted in PubMed, PubMed Health, and PubMed Central, Scopus, and ScienceDirect.

Search terms were based on a Centers for Disease Control and Prevention (CDC) list of general resources related to possible health concerns associated with tsunamis and the World Health Organization (WHO) document “Communicable diseases following natural disasters: risk assessment and priority interventions” [18]. A list of known respiratory pathogens was compiled and used to generate key search terms for the identification of RIDs.

Combinations of search terms for both RIDs and earthquake-induced tsunamis were used to create several search strategies and find relevant results in the online databases. All papers with the specified search terms in their titles, abstracts, or key words were searched for.

The grey literature was also thoroughly studied, and an online search using related key words and their combination was undertaken using Google advanced search and Google Scholar for incorporating scientific journal articles and official reports not included in the aforementioned databases.

Inclusion criteria were as follows: (a) Literature type: published articles and official reports in English; (b) natural disaster: earthquake-induced tsunamis; (c) population: human; and (d) outcome measure: RID incidence increase or outbreak. To avoid excluding potentially relevant studies of public health importance, no standard definition of what constituted an outbreak was used. Instead, studies were included in the review if they provided data for increased incidence of RIs. In addition to being inclusive of a diversity of studies, no filters for identification of specific study design were used. Exclusion criteria were as follows: (a) literature type: news articles, (b) natural disaster: events other than earthquake-induced tsunami, (c) population: non-human, (d) outcome measure: other than RID occurrence and outbreaks.

## 3. Results

### 3.1. Study Selection

**Figure 1:**
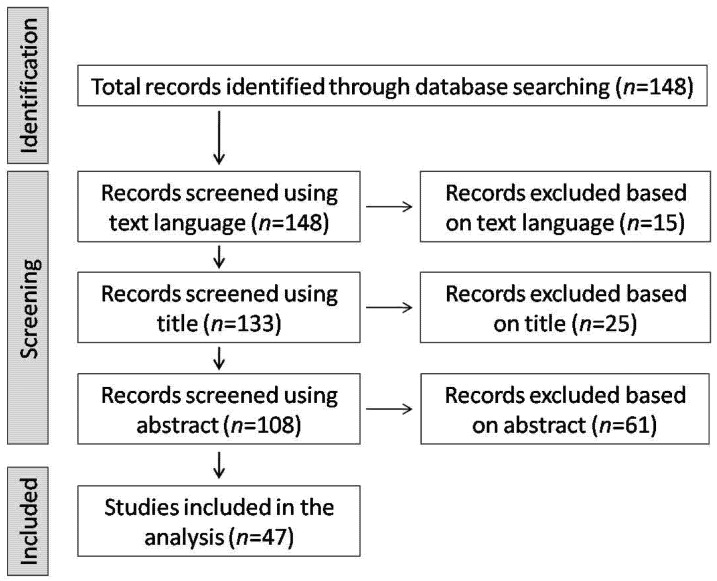
The study selection diagram showing the flow of documents through the literature review.

The initial search generated 148 relevant articles. Fifteen studies were not available in English. Thus, 133 articles were scanned based on their title, and a total of 108 articles were processed in more detail for eligibility. After the full text screening, a total of 47 articles were found to fit the inclusion criteria and were included in the analysis. The following study selection flow-diagram presents our results in detail (Figure 1).

**Figure 2:**
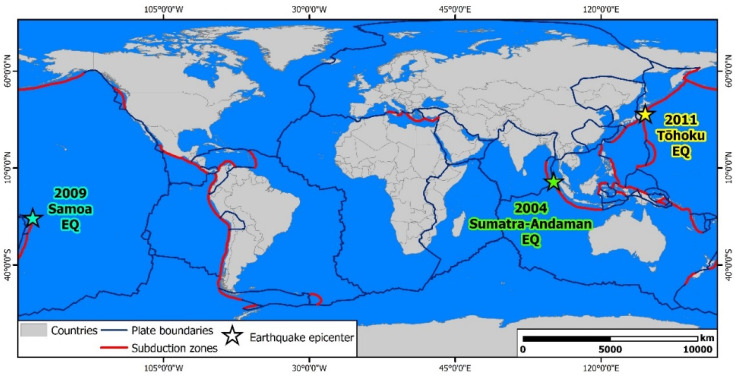
Epicenters of the earthquakes that caused the most devastating tsunami in recent history. More specifically, the Mw = 9.2, 26 December 2004 Sumatra—Andaman earthquake triggered the Indian Ocean tsunami, the Mw = 8.1, 29 September 2009 Samoa earthquake triggered the Samoa tsunami and the Mw = 9.0, 11 March 2011 Tōhoku (Japan) earthquake generated the Great East Japan tsunami.

After the completion of the literature search and the application of the inclusion criteria, the analyzed published articles referred to the public health impact of the three most devastating tsunami induced by great earthquakes in the Indian and Pacific Oceans covering the period from 2004 to 2011. In particular, articles referred to the Indian Ocean tsunami induced by the Mw = 9.2, 26 December 2004 Sumatra—Andaman earthquake, the Samoa tsunami induced by the Mw = 8.1, 29 September 2009 Samoa earthquake, and the Great East Japan tsunami induced by the Mw = 9.0, 11 March 2011 Tōhoku (Japan) earthquake (Figure 2).

### 3.2. The Studied Tsunami

#### 3.2.1. The 2004 Indian Ocean Tsunami

On 26 December 2004, at 07:58:50 local time (00:58:53 GMT), a powerful undersea earthquake of magnitude Mw = 9.2 that struck off the west coast of Sumatra island, Indonesia set off the 2004 Indian Ocean tsunami (also known as the Christmas or Boxing Day tsunami). The Sumatra–Andaman earthquake was caused by a rupture along the fault between the Indo-Australian plate and the southeastern portion of the Eurasian plate. As a result, the sea floor uplifted by several meters leading to the generation of the most powerful and destructive tsunami in modern times and the deadliest tsunami-related disaster in recorded history, with more than 283,000 fatalities (including those who perished and are still missing) and devastation in 14 countries around the Indian Ocean and especially throughout the Bay of Bengal [19,20].

#### 3.2.2. The 2009 Samoa Tsunami

On 29 September 2009, at 06:48 local time (17:48:10 GMT) a large earthquake of magnitude Mw 8.1 struck off-shore of the Samoa Islands in the southern Pacific Ocean adjacent to the Kermadec-Tonga subduction zone. A tsunami was generated that reached the Samoa Islands in about 15 to 20 min and resulted in 189 fatalities (146 in Samoa, 34 in American Samoa, and 9 in Tonga) [21,22].

#### 3.2.3. The 2011 Great East Japan Tsunami

On 11 March 2011, a great earthquake of Mw = 9.0 occurred in the Pacific Ocean, just off the coast of Tōhoku, Japan. This earthquake, also known as the Great East Japan earthquake, occurred at the margin of the Eurasian plate and the Pacific plate. The vertical uplift on the sea-floor at the epicentral area was estimated to be more than 4.5 m, while to the west, both offshore and onshore, a subsidence of 1–1.5 m was observed [23]. The generated tsunami waves traveled across the entire Pacific Ocean, but their effects were devastating to the coastal areas of Northeast Japan, striking the areas between Aomori Prefecture (to the north) and Chiba Prefecture (to the south), a distance of about 850 km [24].

It was the most powerful earthquake ever recorded to have hit Japan, and the fourth most powerful earthquake globally since 1900, resulting in 15,893 fatalities, 6152 injured and 2556 people missing across 20 prefectures of the Eastern Japan based on official reports [25]. Apart from damage on building stock and infrastructures, the tsunami devastated the Fukushima Daiichi Nuclear Power Plant complex leading to extended evacuation of almost 150,000 residents from prohibited access and on-alert areas according to the evacuation process developed by Japan authorities.

### 3.3. Respiratory Infectious Diseases Following Earthquake-Induced Tsunami

Of the 47 articles identified, 26 (55.3%) reported on the occurrence of RIDs after the 2011 Great East Japan tsunami, followed by 20 studies (42.6%) referring to the 2004 Indian Ocean tsunami and only one (2.1%) to the 2009 Samoa tsunami. It is demonstrated that the devastating effects of the tsunami and the harsh post-tsunami conditions have favoured the RID emergence and incidence increase, which are thoroughly described below (Table 1 and Table 2).

#### 3.3.1. Tsunami Lung

Drowning and near-drowning are common during and after disaster-related flooding [26]. People being swept and submerged by tsunami waves are forced to inhale salt water contaminated with soil, sand, and sewer material [27]. The pathophysiology of tsunami-associated aspiration pneumonia, known as “tsunami lung”, involves not only mechanical or chemical-induced lung inflammation, but also bacterial infection induced by intrapulmonary inoculation of various bacteria isolated from saltwater, freshwater, and soil [28].

In Thailand, in the aftermath of the 2004 Indian Ocean tsunami, several campaigns were launched to control emerging infectious diseases, such as food-, water-, animal-, and vector-borne diseases, and respiratory infections in the affected area [29]. An epidemic peak of admitted pneumonia was detected after the tsunami when the data four weeks before the tsunami and four weeks after were further analyzed [29]. The tsunami lung was present in many survivors from the Indian Ocean tsunami and was proposed to be an important infectious disease occurring after the tsunami disaster [29]. The Singapore Armed Forces Medical Team provided primary health care to 2183 tsunami survivors from two locations within Banda Aceh, Indonesia and demonstrated that approximately one-third of the patients suffered from respiratory tract infection, some due to aspiration of sea water [30].

In the case of medical infrastructure destruction due to tsunamis, pulmonary infections are often not diagnosed early and treated effectively, resulting in chronic pyogenic lung disease and necrotizing pneumonia. Additionally, microbes multiply rapidly, enter the bloodstream, and reach the central nervous system, where they produce brain abscesses and neurological problems, such as paralysis [31]. A 17-year-old girl aspirated water and mud while engulfed by 2004 tsunami waves. She developed pneumonia and hemiparesis. A chest radiograph revealed hydropneumothorax and a computed tomography brain scan showed four abscesses, confirming the diagnosis of tsunami lung [31].

Microbiology for the upper and lower respiratory tract reveals a variety of common, but also uncommon pathogens, including a substantial number of highly resistant species. According to reports from Thailand, Finland, Germany, Italy, and Sweden, severely injured European tourists repatriated to their native countries following the tsunami and had polymicrobial infections, most often with *Aeromonas* species and enteric gram-negative bacilli. Gram-positive bacteria including *Staphylococcus*, *Enterococcus*, and *Streptococcus* species were less commonly detected [32,33,34,35,36,37,38]. Except from the abovementioned bacteria, coexistent contamination with fungi and atypical mycobacteria was also observed [36,38,39].

A 35-year-old man injured during the tsunami in Southeast Asia on 26 December 2004 had inhaled seawater when he nearly drowned. He had sinus discomfort but no difficulty breathing. Culture of material obtained from the maxillary sinuses showed *Aeromonas veronii*, *Klebsiella pneumoniae*, *Escherichia coli*, *A. hydrophila*, and *Proteus mirabilis* [40].

After the 2009 Samoa tsunami, 29 patients with respiratory symptoms and a history of aspirating contaminated seawater were diagnosed with aspiration pneumonia [27]. *Streptococcus* spp., *Pseudomonas aeruginosa*, *Citrobacter* spp., *Proteus* spp., *Klebsiella* spp., *Pantoea* spp., and *Enterobacter* spp. were isolated from single or polymicrobial sputum cultures [27]. In comparison to the 2004 Indian Ocean tsunami, infections due to atypical mycobacteria and fungi were not reported.

A 74-year-old man who survived the tsunami after the Great East Japan Earthquake developed aspiration pneumonia and pleural empyema of the right thorax. The causative organisms of pleural empyema were *Streptococcus sanguinis* and *S. mitis*. These viridans streptococci are generally found in dental plaque that, in this case, was inadvertently swallowed and adhered to a pine tree branch, a foreign body during the near-drowning event [41]. Additionally, a 31-year-old woman, injured by the 11 March 2011 tsunami, developed tsunami sinusitis and the culture of sinus contents showed *P. aeruginosa*, *Proteus vulgaris*, and *E. coli* [42].

Inoue et al. (2012) reported on three female patients who later died and suffered from severe lung disorders caused by near-drowning during the 2011 Great East Japan Tsunami. Sputum bacterial culture yielded isolation of *Stenotrophomonas maltophilia*, *Legionella pneumophila*, *Burkholderia cepacia*, and *P. aeruginosa* [28].

Melioidosis is caused by *Burkholderia pseudomallei* and is usually acquired by wound contamination, inhalation, and near-drowning aspiration. The clinical manifestations may range from asymptomatic to acute sepsis with a fatal outcome [43]. Except from the occurrence of multiple abscesses in skin and soft tissue, the bacterial dissemination to distant sites usually results in mild or severe pneumonia and hepatosplenic abscesses. Melioidosis was considered as a potential complication of the Indian Ocean tsunami. *B. pseudomallei* isolation from people injured or who almost drowned in Aceh Province, Indonesia and Phuket Province, Thailand has been reported [11,32,34,35,37,44,45].

Three Finnish tourists visiting the southwest coast of Thailand were found to be infected with *B. pseudomallei* upon their return to Finland [35]. The lengthy incubation period between exposure and melioidosis occurrence demonstrates the importance of clinical vigilance to detect new cases that can occur several days after the tsunami [46]. Athan et al. reported on four patients with culture-confirmed melioidosis after near-drowning in contaminated saltwater and confirmed the existence of *B. pseudomallei* in the Aceh Province, Indonesia [44]. Six melioidosis cases associated with aspiration were reported in tsunami survivors admitted to Takuapa General Hospital in a southern Thailand region where melioidosis is not endemic [34]. This study indicated that tsunami survivors were at increased risk of melioidosis if they were injured in areas where high *B. pseudomallei* prevalence was observed.

The volunteer medical team at Rajavithi Hospital in Bangkok, Thailand treated 37 patients who had aspirated saltwater contaminated with soil and had soft-tissue infections. Among them, one human immunodeficiency virus (HIV)-positive patient and one patient with diabetes mellitus developed lung abscesses and acute lobar pneumonia, respectively, due to *B. pseudomallei* [11].

Fungal infections are important complications in patients after near-drowning [47,48,49,50]. *Scedosporium apiospermum*, the asexual form of *Pseudallescheria boydii*, is a ubiquitous saprophytic filamentous fungus present in soil, manure, sewage, and polluted waters. In near-drowned tsunami victims, *Scedosporium* conidia entering through the respiratory tract may produce necrotizing pneumonia due to spore germination and hyphal invasion. Once in the bloodstream, fungi disseminate to several sites (kidneys, thyroid, and eyes) but develop mainly in the central nervous system, causing granulomata or abscesses and neutrophilic meningitis [48,51]. Nakamura et al. (2011) detected lung and brain abscesses caused by *S. apiospermum* in a 59-year-old Japanese woman who survived the northeastern Japan tsunami [48]. Two years later, the same research group reported the detection of *S. aurantiacum* in the respiratory tract of a 68-year-old Japanese woman who was also washed away by the tsunami [52]. Additionally, three near-drowned individuals during the 2004 tsunami were infected by different species of the *P. boydii* complex [47].

A previously healthy, 59-year-old Swiss male tourist was treated for severe atypical infection upon his return home. He presented with polymicrobial aspiration pneumonia due to multidrug-resistant *Acinetobacter baumannii* and *E. coli*. One month after his discharge from hospital, the patient returned with spondylodiscitis caused by *S. apiospermum* [38]. Additionally, Shimizu et al. (2014) reported on a rare case of a fungal vertebral osteomyelitis due to *S. apiospermum* in a 45-year-old male tsunami survivor of the Great East Japan Earthquake. The infection extended from his lungs to the intervertebral disc and vertebral bodies, and the patient developed severe back pain [49]. According to Igusa et al. (2012), a 73-year-old woman presented with *E. coli* pneumonia in combination with fungal sinusitis and meningitis after the Great East Japan Earthquake and the associated tsunami disaster [50].

Except for *P. boydii*, *Aspergillus fumigatus* has also been implicated as a causative agent of tsunami lung. After the 2011 Great East Japan Earthquake and tsunami, a previously healthy near-drowned 68-year-old female victim who later died was diagnosed with pneumonia caused by *A. fumigatus*. Upon autopsy, evidence of multi-organ disseminated aspergillosis was found [53].

**Table 1:** Included studies referring to the occurrence of tsunami lung clustered by event and disease/pathogen reported. 1: the 2004 Indian Ocean tsunami, 2: the 2009 Samoa tsunami, 3: the 2011 Great East Japan tsunami.

Source	Tsunami	Patients	Clinical Presentation—Causative Pathogens
[11]	1	37 patients	Aspiration pneumonia (*n* = 17), pneumothorax (*n* = 7), pneumomediastinum (*n* = 3), *B. pseudomallei* (*n* = 2)
[29]	1		Epidemic peak of admitted pneumonia, tsunami lung was present in many survivors from the Asian tsunami
[30]	1	325/1021 patients (32%) 394/1162 patients (39%)	Upper respiratory tract infections (mild cough, sore throat), some due to aspiration of sea water
[31]	1	17-year-old female	Pneumonia and hemiparesis (hydropneumothorax and brain abscesses)
[32]	1	26 tsunami victims	Pneumothorax/pneumomediastinum (*n* = 5), bacterial pneumonia (*n* = 18) Aerobic gram negative bacteria (*n* ≤ 9) and *B.pseudomallei*(*n* = 2), 2 deaths
[33]	1	22-year-old Thai male 29-year-old Thai female 30-year-old Thai female	Dyspnea from aspiration pneumonia Dyspnea from aspiration pneumonia Bilateral infiltration in both lower lung fields (gram negative microorganisms)
[34]	1	6 patients	Melioidosis, *B. pseudomallei*
[35]	1	47 year old Finnish male	Melioidosis, *B. pseudomallei*
[36]	1	17 German patients (10 females, 7 males)	Severe large-scale soft-tissue damage: highly resistant bacterial species, fungi and moulds Pneumonitis and pneumonia: multiply resistant *Acinetobacter baumanii* (*n* = 3), multiply resistant *Enterococcus faecium*, sensitive to glycopeptides only, *K. pneumoniae*, intermediate sensitive to amikacin only, MRSA, sensitive to fosfomycin, rifampicin, linezolid and glycopeptides only, and *S. maltophilia*, sensitive to quinolones only) Sinusitis (*n* = 3-multiply resistant *A. baumanii*, intermediate sensitive to ampicillin/sulbactam only, *E. faecium*, sensitive to glycopeptides only, and *C. albicans*, *n* = 1-*A. fumigatus*)
[37]	1	72-year-old Italian female	Melioidosis, *B. pseudomallei*
[38]	1	59-year-old Swiss male 51-year-old Swiss female	Aspiration pneumonia (*A. baumannii*, *E. coli*), spondylodiscitis (*S. apiospermum)* Soft-tissue wounds (*A. baumannii*, *S. maltophilia*, *Achromobacter xylosoxidans*, *E. faecium*, *P. aeruginosa*), pneumonia (*Pseudomonas* sp.), brain abscess (*S. apiospermum*)
[40]	1	35-year-old male	Tsunami sinusitis (*A. veronii*, *K. pneumoniae*, *E. coli*, *A. hydrophila*, *P. mirabilis*)
[44]	1	4 patients	Melioidosis, *B. pseudomallei*
[45]	1	62-year-old female 6–10 patients	Persistent cough, dyspnea and weakness, fever, necrotizing pneumonia Fluctuating fever, chronic, non-productive cough, bilateral, asymmetric, necrotizing pneumonia with cavitation (*B. pseudomallei*: pleural fluid of 2 of these patients, *Nocardia* sp.: sputum of 1 of these patients).
[47]	1	3 patients	Tsunami lung, *Pseudallescheria boydii*
[27]	2	29 patients	Aspiration pneumonia (*Streptococcus* spp., *Pseudomonas aeruginosa*, *Citrobacter* spp., *Proteus* spp., *Klebsiella* spp., *Pantoea* spp., *Enterobacter* spp.)
[41]	3	74-year-old male	Aspiration pneumonia and pleural empyema (*Streptococcus sanguinis*, *S. mitis*)
[42]	3	31-year-old female	Tsunami sinusitis (*Pseudomonas aeruginosa*, *Proteus vulgaris*, *Escherichia coli*)
[28]	3	3 female patients	Severe lung disorders (*S. maltophilia*, *Legionella pneumophila*, *Burkholderia cepacia*, and *P. aeruginosa*), 3 deaths
[48]	3	59-year-old Japanese female	Lung and brain abscesses, *S. apiospermum*
[49]	3	45-year-old male	Tsunami lung, vertebral osteomyelitis (*S. apiospermum*)
[50]	3	73-year-old female	Pneumonia (*E. coli*), fungal sinusitis and meningitis
[52]	3	68-year-old Japanese female	Tsunami lung, *S. aurantiacum*
[53]	3	68-year-old female	Pneumonia (*A. fumigatus*)

#### 3.3.2. Acute Respiratory Infections

Epidemics of acute respiratory infection (ARI) have often been reported in crisis-affected populations [54]. After the 2004 Indian Ocean and the 2011 Great East Japan tsunami, ARI was a prevailing infectious disease among survivors in evacuation shelters [55,56].

Among the 4710 patients, receiving medical treatment by two Korean medical relief teams in southern Sri Lanka in response to the tsunami disaster in South Asia, 1374 (29.2%) displaced individuals presented with trauma-related illnesses, while 1310 (27.8%) presented with acute respiratory infections [57].

Kawano et al. (2016) studied the medical records of evacuees from shelters in Ishinomaki city, Japan, and confirmed that shelter overcrowding was associated with an increased ARI incidence rate compared to non-crowded shelters during the three-week period after the 2011 tsunami occurrence [58].

In Aceh Province, ARI cases increased eight-fold following the 2004 Indian Ocean tsunami. A large percentage of cases occurred in adults, with less than one-third in children under 5 years of age. It is worth mentioning that the number of ARI cases decreased significantly within a month after the disaster event [55,59].

Apart from children <5 years old, elderly and immunocompromised individuals are at increased risk of ARIs [54]. Japan is one of the most rapidly aging societies in the world [60]. Yamanda et al. (2013) reported a substantial increase in the percentage of elderly patients hospitalized for respiratory disease after the Great East Japan tsunami, in comparison to the previous two years (2009–2010). Pneumonia, acute exacerbation of chronic obstructive pulmonary disease (AE-COPD), and asthma attacks were more common after the disaster [61]. According to Suzuki et al. (2011), pneumonia in older evacuees may have resulted from impaired oral hygiene, frequent aspiration, malnutrition, and cold temperatures under favorable circumstances [62].

- Bacterial pneumonia

Community-acquired pneumonia (CAP) by *Streptococcus pneumoniae*, *Haemophilus influenza*, and *Moraxella catarrhalis* comprised 43% of hospital admissions for infectious disease one month after the 2011 Great East Japan tsunami [63]. At the same time, a survey was carried out on disease prevalence changes among inpatients in respiratory medicine departments of regional core hospitals in the Miyagi Prefecture. The number of inpatients from 11 March to 10 April 2011 was 2.7 times greater than the number admitted during the same period in 2010. Additionally, the number of patients diagnosed with CAP was 2.2 times greater in 2011 than in 2010 [64].

During the three-month period following the disaster, Daito et al. (2013) observed a significant increase not only in the weekly incidence rates of pneumonia hospitalizations, but also in pneumonia-associated deaths, especially among nursing home residents, most of whom were elderly people (aged ≥ 80 years) with physical and mental limitations who needed assistance with daily activities [65]. Shibata et al. (2016) examined the association between disasters and pneumonia death, and revealed that the 2011 Great East Japan Tsunami increased the pneumonia death risk in addition to the effects of the earthquake, especially in the coastal municipalities compared to the inland ones of Miyagi, Iwate, and Fukushima Prefectures [66]. According to Takahashi et al. (2012), the number of pneumonia cases, mainly caused by *H. influenzae* and *M. catarrhalis*, peaked in the first three weeks after the 2011 Great East Japan earthquake, followed by a gradual decrease after the fourth week. On the contrary, pneumonia caused by enterobacteria, staphylococci, or atypical pathogens showed no increase after earthquakes [67]. Watanabe et al. (2007) confirmed that ARIs caused by various types of *H. influenzae* and *S. pneumoniae* were prevalent and potentially transmitted among internally displaced persons in three evacuation camps in Sri Lanka [68].

- Legionellosis

*Legionella pneumophila* causes human legionellosis that varies in severity from an acute, self-limited, febrile illness, known as Pontiac fever, to severe and sometimes fatal pneumonia accompanied by multisystemic disease [69]. This gram-negative bacillus is usually found in moist soil and water in lakes, rivers, swamps, and hot springs. It is usually transmitted by aerosolization or aspiration of contaminated water. Since tsunami waters contain soil microbes, the possibility of legionellosis must be considered when examining patients suffering from tsunami-associated pneumonia.

Ebisawa et al. (2011) reported pulmonary co-infection with Legionella and multiple antibiotic-resistant *E. coli* in a previously healthy 75-year-old woman, as a result of immersion in tsunami waters 1 km inland from the Pacific coastline after the 2011 Great East Japan earthquake [70]. Nakadate et al. (2012) reported on two tsunami victims, a 33-year-old woman and a 2-year-old girl, that were engulfed by the tsunami in Rikuzentakada city, Iwate prefecture, Japan and developed severe pneumonia potentially caused by *L. pneumophila*. The adult patient presented massive hemoptysis and died because of airway occlusion and hypotension. Although the girl’s symptoms improved gradually, the outcome of the illness was fatal since the girl died from brainstem hemorrhage caused by a mycotic intracranial aneurysm [71].

- Influenza infection

The influenza virus is one of the most common causes of ARIs that are usually mild and self-limited, but can lead to severe complications, such as pneumonia, myocarditis, and influenza-associated encephalopathy, especially in vulnerable groups of people such as the elderly, young children, pregnant women, and individuals with underlying health conditions [72,73].

At the time of the Great East Japan Earthquake on 11 March 2011, the influenza A (H3N2) virus was the predominant subtype circulating in the Miyagi Prefecture. Thus, there was an increased concern about the profound impact of severe influenza A (H3N2) outbreaks, especially upon the elderly, representing the majority of evacuees in evacuation centers (ECs) [74,75,76]. Post-tsunami outbreaks of influenza A occurred at ECs in Miyagi Prefecture and subsided with no flu-related deaths [73,77]. In Kesennuma and Natori ECs, evacuees were exposed to cold, unhygienic conditions, as well as malnutrition due to power failures, lack of running water, and insufficient food supplies [77]. Additionally, 105 confirmed influenza A cases were reported from 23 March to 11 April 2011, mostly in evacuees aged 15–64 years in five evacuation centers in Yamamoto town, whereas no influenza B infection cases were recorded [73]. The beginning of the influenza outbreak more than one week after the earthquake and its simultaneous spread to different ECs led Kamigaki et al. (2014) to suggest that the influenza virus was introduced into the ECs through search and rescue activities, when relief workers were engaged with individuals arriving from affected areas [73].

During March–May 2011, influenza A (H3N2) and influenza B viruses were detected in samples from patients with suspected influenza infection in Miyagi Prefecture. Phylogenetic analysis revealed different clades of the influenza A (H3N2) virus before and after the earthquake [78]. Although the influenza A (H3N2) virus was the dominant virus until the 14th week, a sudden increase in the number of influenza B cases occurred after schools reopened. The Yamagata lineage of influenza B was detected in one patient from western Japan, indicating that the source of influenza infection is not only a microorganism circulating in the community but also, though even less likely, one introduced into the affected area [78].

After the Great East Japan Earthquake and Tsunami of 11 March 2011, approximately 1800 survivors were placed in an evacuation shelter, called the K-wave gymnasium. From 21 March to 37 March 2011, 15 individuals were found positive for influenza A. Namiki et al. (2013) indicated that the design of the K-wave gymnasium and the separation of the patients from other evacuees prevented any further spreading of the influenza epidemic in this facility [79].

- Measles

The measles virus (MV) is highly contagious and spread by coughing and sneezing, and close personal or direct contact with infected nasal or throat secretions. Measles transmission was observed in population affected by the 2004 Indian Ocean tsunami and was dependent on the baseline vaccination coverage rates among the affected population, especially among children aged <15 years [80,81]. Following the 2004 tsunami disaster in Banda Aceh, Indonesia, a cluster of 35 measles cases occurred in a susceptible community living in unplanned and crowded camps in the Aceh Utara district, while sporadic cases were common despite mass vaccination campaigns [82]. Between December 2004 and February 2005, Mohan et al. (2006) detected 101 measles cases originating not only from tsunami-stricken, but also from unaffected areas in Tamil Nadu, India. The median ages of case-patients in both areas were ≤5 years. According to this study, measles transmission was not related to the tsunami and continued despite high one-dose measles vaccine coverage in the above-mentioned areas [80].

- Tuberculosis

Tuberculosis (TB) is a multisystemic disease with various manifestations, caused by *Mycobacterium tuberculosis*. TB is spread from person to person through the air, when people with lung TB cough, sneeze, or spit. When an individual develops active TB disease, the symptoms may be mild for many months, leading to delays in seeking care and transmission of the bacteria to others. The diagnosis and treatment of latent tuberculosis infection (LTBI) have become mandatory to reduce the burden of tuberculosis worldwide.

Kanamori et al. (2016) investigated clinical characteristics and prognosis in TB patients and the transmission dynamics of TB after the 2011 Japan earthquake and tsunami. Risk factors for TB prognosis were associated with advanced age, low serum albumin level, functional status at admission, and oxygen requirement. Most of the cases with pulmonary TB experienced reactivation of latent TB infection [83]. According to Sakurai et al. (2016), the numbers of tuberculosis patients and patients with LTBI significantly increased in the post-disaster period compared to the pre-disaster one, especially among evacuees staying in crowded shelters in coastal regions of the northern Miyagi Prefecture, Japan [84]. Othman et al. (2007) reported on two patients who survived near-drowning episodes during the Sumatra-Andaman tsunami and were infected with both TB and melioidosis and the second patient with *Salmonella typhi* as well [85]. Apart from melioidosis, a well-known complication of the December 2004 tsunami in South East Asia, the awareness of local health authorities should be raised regarding a possible increase in the incidence of TB in tsunami-affected areas.

**Table 2:** Included studies referring to the occurrence of acute respiratory infections clustered by event and disease/pathogen reported. 1: the 2004 Indian Ocean tsunami, 3: the 2011 Great East Japan tsunami.

Source	Tsunami	Patients	Clinical Presentation—Causative Pathogens
[55]	1	37,492 ARIs cases (WHO) during first five months after the tsunami	The highest percentage of ARI cases occurred within 2 months after the 2004 tsunami.
[57]	1	4710 patients in southern Sri Lanka	1374 (29.2%) patients: trauma-related illnesses 1310 (27.8%) patients: ARIS
[68]	1	324 internally displaced persons in 3 different tsunami disaster evacuation camps of Sri Lanka	ARIs caused by various types of *H. influenza* and *S. pneumoniae* were prevalent and some of them, including resistant isolates, were potentially transmitted from person to person in tsunami disaster evacuation camps in Sri Lanka.
[80]	1	101 measles cases	Measles virus circulated in Cuddalore district following the tsunami, although there was no association between the two events.
[82]	1	35 measles cases	The cluster occurred in a susceptible community living in unplanned and crowded camps in Aceh Utara district, Indonesia
[85]	1	2 patients	Multiple infection (tuberculosis and melioidosis)
[56]	3	1167 patients, 6 shelters	Outbreaks of ARI and acute gastroenteritis occurred in evacuation shelters.
[58]	3	7439 patients from 44 shelters	Increased ARI incidence rate in crowded shelters
[61]	3	322 patients with respiratory diseases (11 March–9 May 2011), 99 and 105 patients (corresponding periods in 2009 and 2010)	Increase in the absolute numbers of admissions was highest for pneumonia, followed by acute exacerbation of chronic obstructive pulmonary disease (AE-COPD) and asthma attacks
[62]	3	17 individuals	Pneumonia in older refugees (possible causes: impaired oral hygiene, frequent aspiration, undernutrition, cold temperatures under unfavorable circumstances)
[63]	3	1577 patients	Pneumonia comprised 43% of cases (*Streptococcus pneumoniae*, *Moraxella catarrhalis*, *Haemophilusinfluenzae*)
[64]	3	inpatients in respiratory medicine departments of regional core hospitals in Miyagi Prefecture	The number of patients diagnosed with CAP was 2.2 times greater in 2011 than in 2010
[65]	3	A total of 550 pneumonia hospitalizations were identified, including 325 during the pre-disaster period and 225 cases during the post-disaster period.	A marked increase in the incidence of pneumonia was observed during the 3-month period following the disaster. Leading causative pathogens: *S. pneumoniae*, *H. influenzae* and *K.pneumoniae*. The positivity of *H.influenzae* increased by 4-fold after 11 March, especially among patients from evacuation shelters.
[66]	3	6603 participants died of pneumonia during 1 year after the earthquake.	An earthquake increased the risk of pneumonia death and tsunami additionally increased the risk.
[67]	3	49 adults with pneumonia (controls): within 6 weeks before the earthquake 172 adults with community-acquired or health care-associated pneumonia: within the 9 weeks after the earthquake	The number of patients with pneumonia peaked in the first 3 weeks after the earthquake, followed by a gradual decrease starting from 4 weeks after the earthquake. *H. influenzae* and *M. catarrhalis* were more predominant than *S. pneumoniae*
[70]	3	75-year-old female	Pulmonary co-infection with Legionella and multiple antibiotic-resistant *E. coli*
[71]	3	33-year-old female 2-year-old female	Severe pneumonia potentially caused by *L. pneumophila*, 2 deaths
[73]	3	105 confirmed influenza cases in five ECs	An outbreak of influenza A (H3N2) occurred in the ECs after the Great East Japan Earthquake of 2011
[77]	3	25 patients diagnosed with influenza (Kesennuma City Gymnasium, Kesennuma) 20 individuals diagnosed with influenza (Tatekoshi Elementary School, Natori)	Two post-tsunami outbreaks of influenza A in evacuation centers in Miyagi Prefecture, Japan
[78]	3	277 samples tested for influenza virus from Sendai City and evacuation centers in Miyagi Prefecture	Influenza A (H3N2) (*n* = 112 cases), influenza A (H1N1) 2009 (*n* = 1 case), influenza B (*n* = 92 cases)
[79]	3	15 individuals found positive for Influenza A in the Kesennuma City General Gymnasium (K-Wave)	The design of the K-wave gymnasium and the separation of evacuees from the patients prevented any further spreading of the influenza epidemic
[83]	3	93 pulmonary TB patients (tsunami-affected areas 25, non-tsunami areas 68)	Risk factors for prognosis of TB after the earthquake: advanced age, low serum albumin level, functional status at admission, and oxygen requirement. Most of the cases with pulmonary TB experienced reactivation of latent TB infection
[84]	3	Monitoring of TB and LTBI patients in coastal and inland shelters of Northern Miyagi Prefecture, Japan	The numbers of TB patients and of patients with LTBI significantly increased in the post-disaster period, especially among evacuees staying in crowded shelters in coastal regions of Northern Miyagi Prefecture

## 4. Risk Factors for Emergence and Transmission of RIs and Lessons Learned for Disaster Risk Management

Outbreaks of infectious diseases often become a public health concern, after disasters induced by natural hazards. The destruction of local health care infrastructure along with insufficient emergency and preparedness plans can compromise the prompt management and effective treatment of severe health problems and contribute to the emergence and rapid spread of infectious diseases.

This study involved an extensive review of the literature related to the impact of the three most devastating tsunamis of the last 20 years induced by great earthquakes, namely the 2004 Indian Ocean tsunami triggered by the Mw = 9.2, 26 December 2004 Sumatra—Andaman earthquake, the 2009 Samoa tsunami generated by the Mw = 8.1, 29 September 2009 Samoa earthquake, and the 2011 Great East Japan tsunami induced by the Mw = 9.0, 11 March 2011 Tōhoku (Japan) earthquake, on public health, and more specifically to the occurrence and transmission of RIDs.

Respiratory infections following a tsunami are often polymicrobial and tend to form chronic pyogenic lung disease, necrotizing pneumonia, and empyemas [31]. Environmental pathogens, multidrug-resistant bacteria, atypical bacteria, and fungi were commonly detected among survivors of the 2004 Indian Ocean tsunami who experienced near-drowning events, as it is confirmed by the increased number of related scientific articles (16/47, 34%). The absence of immediate medical attention due to limited resources and difficult emergency conditions contributed to the severity of complications [38]. Many disaster victims were repatriated and sought medical treatment in their home country [32,33,34,35,36,37,38,39]. Clinical laboratory scientists were taken by surprise when they recovered multiple microorganisms rarely or never encountered among their local patient population.

Although these infections were also reported after the 2009 Samoa tsunami and the 2011 Great East Japan tsunami in a lower percentage (2.1% and 19.1%, respectively), the disease outcome was dramatically improved due to the early initiation of appropriate antibiotic polytherapy and coordinated management in a timely manner based on pathogens that had already been identified as causative agents of tsunami lung in the 2004 Indian Ocean tsunami [38].

Early diagnosis of fungal infections may be difficult, often resulting in severe neurological disorders or death, even in immunocompetent patients. *S. apiospermum* and *Aspergillus* spp infections, which were mainly recorded after the 2011 Great East Japan tsunami (10.6%) compared to those after the 2004 Indian Ocean tsunami (4.3%), should be highly considered when treating tsunami survivors to administer prompt effective antifungal treatment with promising outcome.

Since disasters induced by natural hazards do not import new diseases to the affected areas, the transmission of an infectious agent can occur if it is endemic to the affected region or if it is introduced into the region where the disaster occurred through search-and-rescue activities. It is worth mentioning that 8 of the 47 scientific articles (17%) referred to aspiration pneumonia caused by *B. pseudomallei* after the 2004 Indian Ocean tsunami, while there was no report of such infection among the tsunami victims of the Tohoku, Japan earthquake in 2011. These findings are expected since melioidosis is a disease of tropical climates, especially in Southeast Asia and northern Australia where it is primarily found. Additionally, although Legionella and Influenza infection cases (6.4% and 8.5% respectively) were detected following the 2011 Great East Japan tsunami, there was no report of these infections after the Sumatra-Andaman earthquake in 2004. The different spectrum of pathogens isolated from victims residing in different tsunami-affected areas reflects the environmental factors present at the time of the tsunami impact and confirms the observation that infectious diseases already endemic in a particular area may grow into an outbreak when environmental conditions become favorable.

Near-drowned victims admitted to hospitals with respiratory symptoms were mainly diagnosed with tsunami-associated aspiration pneumonia, while increased ARI incidence rate was recorded among survivors in evacuation centers. In Japan, the increase of hospitalization due to RDIs was observed among adults of all age groups, indicating that this situation was favored not only by ageing, but also by other factors shared by all survivors. The overcrowding of emergency shelters combined with inadequate air ventilation, impaired oral hygiene and poor nutrition led to the development of ARIs especially among older evacuees.

Additionally, hypothermia and psychological stress played a decisive role. On 11 March 2011, it was snowing in northern Miyagi. After the tsunami disaster, the majority of the evacuation shelters were not sufficiently equipped with heating sources and it is well-known that hypothermia can increase the risk of subsequent infections, including pneumonia [86,87]. Tsunami survivors experienced stress reactions after the disaster, which may weaken the immune system and increase the risk of respiratory infections [88,89].

Influenza can have a significant public health impact if a disaster induced by natural hazard occurs during the transmission period. At the time of the Great East Japan earthquake on 11 March 2011, the influenza A (H3N2) virus was the predominant subtype circulating in Miyagi Prefecture. Thus, the possibility of an influenza outbreak was extremely high, as it was confirmed by several reports (8.5%) referring to influenza A outbreaks among evacuees and the internally displaced population. After May 2011, the risk of pneumonia was reduced due to the decline in the evacuees’ number, improvements in living conditions, and recovery of food and medical supplies. *S. pneumoniae*, *H. influenzae* and *M. catarrhalis*, the typical bacterial pathogens that cause CAP, are transmitted person-to-person at day-care centers and within households [90,91,92]. An increase of CAP cases was reported in the first weeks after the 2011 Great East Japan Tsunami and was detected among patients from overcrowded evacuation shelters.

Except for influenza infections and CAP cases, overcrowding conditions in emergency and evacuation shelters and an interruption of public health campaigns for the elimination of vaccine-preventable illnesses can facilitate rapid measles virus transmission, which is dependent on the baseline immunization coverage among the affected populations. Administration of highly effective vaccines, such as MMR (measles, mumps and rubella), flu, *H. influenzae* type b (Hib), and pneumococcal vaccines should be conducted as soon as people start gathering in camps, following CDC and WHO recommendations to reach and sustain higher immunization coverage levels, necessary to prevent the occurrence and rampant spread of measles, influenza epidemics, and invasive diseases caused by *H. influenzae* and *S. pneumoniae*.

Moreover, tuberculosis is a growing concern in overcrowded evacuee settings. Lee et al. demonstrated that close contacts of active TB patients were at increased risk of both latent and active tuberculosis infection [93]. Factors such as population displacement, poor access to or breakdown of healthcare services, and interruption of control programs or ongoing treatment may increase disease severity and treatment burdens and contribute to the subsequent high transmission rate of the disease. Long-term observation is required to detect the prevalence of tuberculosis infection, and early and effective preparation of shelters is necessary to avoid overcrowding.

Knowledge of the factors underlying respiratory infection emergence can contribute to the development and implementation of more effective prevention strategies. Effective global and local disease surveillance is a key prerequisite for early warning and protection against emerging infections and potentially uncontrolled disease transmission. The detected risk factors for respiratory infection emergence and outbreaks included destruction of health care infrastructures, low socioeconomic conditions, exposure to high pathogen densities, aggravating post-tsunami weather conditions, regional disease endemicity, overcrowded evacuation shelters, low vaccination coverage, and poor personal hygiene.

## 5. Conclusions

Earthquake-induced tsunamis constitute a major natural hazard that have high potential to cause more fatalities than the causative earthquake itself. Typical examples are the studied tsunamis in Indonesia and Japan. This potential along with the impact of tsunamis on the affected natural and built coastal environments make tsunamis a natural hazard that should not be overlooked. Among other tsunami effects, infectious diseases can occur not only in developing but also in developed industrialized countries, as it was well-shown by the consequences of the Great Eastern Japan Earthquake and tsunami. Unfortunately, developing countries present unstable socioeconomic conditions with shortage of resources and skills that do not contribute to the development and maintenance of proper health surveillance systems and the smooth function of clinical and laboratory facilities.

Disasters following earthquake-induced tsunamis generate mass casualties within a very short time, which are often accompanied by temporary paralysis of the local emergency response and health care services. Respiratory infections are common following disasters induced by natural hazards, particularly among displaced populations, elderly individuals, and young children. Overcrowded evacuation shelters, poor nutrition, and lack of healthcare services are significant risk factors for respiratory infection emergence and transmission in the aftermath of tsunamis.

Lessons learned from these devastating tsunamis should translate into the updating or the development of appropriate planning and response to other natural disasters or complex emergencies that are certain to follow in the near future. Actually, a similar disaster was repeated when a 7.4 magnitude earthquake hit the island of Sulawesi on 28 September 2018, causing the most devastating earthquake to hit Indonesia since 2004.

The establishment of strong disaster preparedness plans characterized by adequate environmental planning, resistant infrastructures, and resilient health care facilities is significant for the early detection, surveillance and control of emerging infectious diseases. Clinicians should be aware of unusual complications and highly-resistant microorganisms that can lead to extensive illness and death in tsunami survivors, who must be considered high-risk patients, even months or years after the natural disaster. Sharing tsunami experiences both locally and internationally is extremely important in order to improve disaster management and help mitigate tsunami-related impacts on public health, especially in tsunami-prone regions.
